# Deletion of α5 integrin in endothelial cells ameliorates age–associated cognitive decline and increases survival in mice

**DOI:** 10.1002/alz.090545

**Published:** 2025-01-03

**Authors:** Gregory W Hall II, Saifudeen Ismael, Gregory J Bix

**Affiliations:** ^1^ Department of Neurosurgery, Clinical Neuroscience Research Center, Tulane University School of Medicine, New Orleans, LA USA; ^2^ Tulane Brain Institute, Tulane University, New Orleans, LA USA

## Abstract

**Background:**

Levels of inflammatory components gradually rise in tissues and blood as we age. This “inflammageing” process is often debilitating and even fatal. Cognitive impairment is one example of inflammageing’s incapacitating nature. Excessive permeation of inflammatory markers in the brain gradually erodes the blood‐brain barrier (BBB) over time. Consequently, enhanced cerebral inflammatory processes contribute to neurological senescence. Neurological inflammageing may be partly due to α5 integrin, a receptor found primarily in brain endothelial cells during brain development, cerebrovascular injury (e.g. stroke), neuroinflammation and neurodegenerative disease (e.g. ALS). We hypothesize that deletion of α5 integrin in endothelial cells helps maintain BBB integrity and leads to improved cognitive performance with age. In the past we have shown that endothelial cell selective α5 knockout (α5‐EC‐KO) mice are more resilient to ischemic stroke. Enhanced stroke recovery in α5‐EC‐KO mice is due to less α5‐related BBB deterioration and subsequently less brain edema and infiltration of peripheral inflammatory cells. Therefore, we used aged α5‐EC‐KO and wild type (WT) litter mate mice to investigate how α5 integrin influences cognitive performance and survival probability over time.

**Methods:**

25‐32‐month‐old male and female α5‐EC‐KO mice and aged‐matched WT mice were used in the study. Cognitive function was analyzed using Y‐maze and open field. Additionally, Kaplan Meier curves were used to assess probability of survival.

**Results:**

For Y maze, results indicate a trend towards greater alteration rate and total alteration for α5‐EC‐KO mice. Open field test shows greater center time and less peripheral time for α5‐EC‐KO. WT and α5‐EC‐KO mice have nearly identical mean speed. Additionally, Kaplan‐Meier curves indicate a greater probability of survival for α5‐EC‐KO mice between the ages of 26 to 30 months. Interestingly, 26‐30 months is the average life expectancy of a lab mouse.

**Conclusions:**

Observations from Y maze and open field tests suggest that α5‐EC‐KO mice have improved spatial memory and less anxiety behavior when compared to aged‐matched WT mice. Moreover, the Kaplan‐Meier curves depict a greater probability of survival for aged α5‐EC‐KO mice. Thus, α5 integrin expression may significantly influence age‐related cognitive decline. Preliminary data suggests that inhibition of α5 integrin may improve symptoms of age‐associated cognitive impairment.

